# A Menace to Texas

**Published:** 1914-11

**Authors:** 


### A Menace to Texas.

Dr. Carrie Weaver Smith, in a recent address before the Dallas County Medical Society, proffered some startling figures in support of her assertion that the menace of the feeble-minded is growing more grave in Texas. The University of Texas is given as authority that the number of feeble-minded in Texas approximates 7000. Dr. Smith estimates the number in Dallas alone at 1000, and that this number is being augmented by a birth rate of 32 per 1000, as compared to 16 per 1000 of normal children. This startling estimate is followed by an equally startling declaration:

"The feeble-minded of Dallas are making necessary your juvenile courts, your United Charities, two-thirds of your county and city jails, three-fourths of your police force, four-fifths of your immoral women, and half of your saloons."

If this is true of Dallas—and no authoritative voice has been raised to disprove the assertion—a similar condition must prevail throughout all of Texas. The problem, then, has become a State matter, and the State should take early steps to solve it. Little, if any, attention has been given to it in the past. But now that it has assumed the aspect of an increasing menace to the citizenship, remedial legislation should be enacted to lessen this peril in future. With special provision for scientific treatment of the feeble-minded and prohibition of marriage, a long step will be taken toward the desired solution of the now pressing problem. Dr. Weaver advocates also compulsory education as an initial saving step, and condemnation of illicit sale of drugs and legal sale of alcohol as contributing causes. As the expression of a physician, Dr. Weaver's declarations are of special and timely importance and should provoke the thinking citizenship to aggressive action. In the interest of posterity, the medical profession of Texas should make concerted endeavor to open the eyes of the State to this growing menace, and could render worthy and effective service by taking the lead in any movement which has for its object removal of a possibility of further increase in the number of feeble-minded. Society, if it were awake, would demand protection. It is the duty of the physician and the press to awaken the sleeper.—*Dallas Evening Journal.*

